# Erratum

**DOI:** 10.1080/17482631.2017.1385178

**Published:** 2017-10-13

A number of articles were erroneously published in the issue 12(1) of *International Journal of Qualitative Studies on Health and Well-being*. Now, these articles are being republished in issue 12(S2). The articles that were republished in the issue 12(S2) are listed below:

Larsson, I. & Jormfeldt, H. (2017). Perspectives on power relations in human health and well-being. *Journal of Qualitative Studies on Health and Well-Being, 12* (S2), doi: 10.1080/17482631.2017.1358581

Håman L., Lindgren, E.-C & Prell, H. (2017). “If it’s not Iron it’s Iron f*cking biggest Ironman”: personal trainers’s views on health norms, orthorexia and deviant behaviours. *Journal of Qualitative Studies on Health and Well-Being, 12* (S2), doi: 10.1080/17482631.2017.1364602

Jonsson, L, Larsson, C., Christina, Berg, C., Korp, P., & Lindgren, E.C. (2017). What undermines healthy habits with regard to physical activity and food? Voices of adolescents in a disadvantaged community. *Journal of Qualitative Studies on Health and Well-Being, 12* (S2), doi: 10.1080/17482631.2017.1333901

Lindgren, E.C., Annerstedt, C., & Dohsten, J. (2017). “The individual at the centre” – a grounded theory explaining how sport clubs retain young adults. *Journal of Qualitative Studies on Health and Well-Being, 12* (S2), doi: 10.1080/17482631.2017.1361782

Lindgren, E.C., & Barker-Ruchti, N. (2017). Balancing performance-based expectations with a holistic perspective on coaching: a qualitative study of Swedish women’s national football team

coaches’ practice experiences. *Journal of Qualitative Studies on Health and Well-Being, 12* (S2), doi: 10.1080/17482631.2017.1358580

Taylor & Francis apologizes for these errors.

